# The effect of tactile augmentation on manipulation and grip force control during force-field adaptation

**DOI:** 10.1186/s12984-020-0649-y

**Published:** 2020-02-11

**Authors:** Chen Avraham, Ilana Nisky

**Affiliations:** 1 Biomedical Engineering, Ben-Gurion University of the Negev, 8410501 Be’er Sheva, Israel; 2 Zlotowski Center for Neuroscience, Ben-Gurion University of the Negev, 8410501 Be’er Sheva, Israel

**Keywords:** Force-field adaptation, Skin-stretch, Sensory augmentation, Manipulation force control, Grip force control

## Abstract

**Background:**

When exposed to a novel dynamic perturbation, participants adapt by changing their movements’ dynamics. This adaptation is achieved by constructing an internal representation of the perturbation, which allows for applying forces that compensate for the novel external conditions. To form an internal representation, the sensorimotor system gathers and integrates sensory inputs, including kinesthetic and tactile information about the external load. The relative contribution of the kinesthetic and tactile information in force-field adaptation is poorly understood.

**Methods:**

In this study, we set out to establish the effect of augmented tactile information on adaptation to force-field. Two groups of participants received a velocity-dependent tangential skin deformation from a custom-built skin-stretch device together with a velocity-dependent force-field from a kinesthetic haptic device. One group experienced a skin deformation in the same direction of the force, and the other in the opposite direction. A third group received only the velocity-dependent force-field.

**Results:**

We found that adding a skin deformation did not affect the kinematics of the movement during adaptation. However, participants who received skin deformation in the opposite direction adapted their manipulation forces faster and to a greater extent than those who received skin deformation in the same direction of the force. In addition, we found that skin deformation in the same direction to the force-field caused an increase in the applied grip-force per amount of load force, both in response and in anticipation of the stretch, compared to the other two groups.

**Conclusions:**

Augmented tactile information affects the internal representations for the control of manipulation and grip forces, and these internal representations are likely updated via distinct mechanisms. We discuss the implications of these results for assistive and rehabilitation devices.

## Background

In everyday interaction with objects, we must concurrently control and sense internally and externally generated forces to control actions, to estimate the mechanical properties of manipulated objects, and to form an internal representation of the environment that can be used to predict the environment dynamics. There are two major force-sensing modalities in our body – kinesthetic and tactile. Kinesthetic information is sensed by tension of the muscles and Golgi tendon organs. Tactile information is sensed at the points of contact with manipulated objects by mechanoreceptors in the skin [1]. During interaction with objects that are held in our hands, we feel external *load forces* that are dependent on the mechanical properties of the object and our movements during interaction with the objects. In response to these load forces, we control manipulation and grip forces. *Manipulation forces* are the forces and torques that we exert against the held object to translate and rotate it. In contrast, *grip forces* stabilize the grasped object to prevent its slippage without resulting in any other motion [2–4]. Previously, studies showed that sensory information can be used differently to form internal representation for manipulation and grip force control [5–7].

In adaptation studies, the internal representation is typically evaluated from the modifications in participants’ movements as a result of exposure to a dynamic perturbation. Throughout the adaptation, the participants adjust to the perturbation, and modify the kinematics and dynamics of their movements to achieve optimal performances according to the task demands [8–11]. Previously, adaptation to a state-dependent force-field perturbations was extensively investigated [11–19]. When initially exposed to this perturbation, the participants experience an error between the predicted and the actual movements and forces. With continued exposure, the participants adapt to the perturbation by building an internal representation of the perturbing forces that is based on state variables (such as position and velocity) [13–16] to produce manipulation forces that will compensate for the state-dependent perturbation, and thus, the error is reduced. With a sudden removal of the perturbation, the participants exhibit aftereffects, which demonstrate the construction of an internal representation that was used for manipulation force control [11]. Another common way to assess the adaptation and the construction of internal representations is by measuring the manipulation forces that participants apply by introducing virtual force channels that constrain the movement to a straight trajectory [17, 18]. Here, we will use this approach to investigate the effect of augmented tactile information on the way participants adapt to force-field perturbation.

Internal representations are also used to adjust the applied grip force to the anticipated external dynamics [20]. Humans use feedforward control to adjust the grip force to the expected slipperiness and load with some additional safety margin [21–23]. In addition, feedback control is used when sensory information is indicating unexpected load or slippage throughout the interaction, casing an immediate increase of the grip force [1, 24]. In the case of uncertainty about the external load, the safety margin (and the baseline grip force) will increase to ensure sufficient gripping regardless of the load force [25]. Accordingly, grip force control is composed of the following components: *baseline* (initial) *grip force*, that is applied without relation to the external load and is used to prevent slippage, and a *modulation grip force* with load force that has two components: (1) *predictive* modulation of grip force – that is related to the prediction we have about the load force, and (2) *reactive* modulation of grip force – that adjusts the grip force to unexpected changes in the load force during the interaction.

How augmented tactile information affects force-field adaptation is poorly understood. In the first (and to the best of our knowledge only) attempt to answer this question, the authors focused on the kinematics of the hand, and showed that the additional tactile information did not affect the trajectories [19]. Consequently, they concluded that tactile information is not used in force-field adaptation. However, different adaptation mechanisms may result in similar kinematics. For example, internal representations are updated in the case of state-dependent and predictable force perturbations [11, 12]. However, co-contraction of the muscles to increase the impedance of the arm, such as during adaptation to uncertain [26, 27] or unstable [28–30] dynamics, or in initial stages of motor adaptation [27], may also reduce path error, and reflexes and feedback gains may also be adapted in certain situations [31–33]. Therefore, a full understanding of integration of tactile and kinesthetic information in force-field adaptation requires quantifying additional aspects of adaptation, such as the control of manipulation and grip force.

Until recently, to understand the integration between kinesthetic and tactile information for manipulation and grip force control, studies used impaired sensory systems by studying neurological patients or by sensory elimination [34–36]. In the recent years, new devices were developed that can stimulate the tactile mechanoreceptors by deforming the skin, and thereby augment the tactile sensation [37]. Using these devices, it was shown that artificial skin deformation can increase the perceived mechanical properties [4, 38–43], possibly due to an increase in the perceived forces, and substitute and augment kinesthetic information in some motor tasks [44–48]. The effect of artificial skin deformation on the integration of kinesthetic and tactile information for the control of grip force was recently examined in the case of a stiffness perception task [39]. However, the integration process of these two information channels during the process of building an internal representation for manipulation and grip force control during adaptation is not yet fully understood.

In the current study, we use a force-field adaptation as a paradigm for examining the integration of kinesthetic and tactile information for building internal representations in the healthy sensorimotor system. We present kinesthetic and tactile stimuli to the participants, and examine in the same protocol the kinematics, the manipulation forces, and the grip forces. To partially decouple between the two information channels, we combined the same state-dependent force-field that was applied by a kinesthetic haptic device with different state-dependent artificial tactile stimuli. Specifically, we exposed participants to a velocity-dependent force-field in three different conditions: (1) with additional skin-stretch in the *same direction* as the applied force-field, (2) with additional skin-stretch in the *opposite direction* to the applied force-field, and (3) without additional tactile information. When a kinesthetic haptic device is held in a precision grip, the forces that are applied also cause a stretch of the skin at the contact between the device and the fingertips, and cause inherent stimulation of the tactile mechanoreceptors. Hence, in these three conditions the participants experienced: (1) augmented tactile stimulation (i.e. the artificial stretch that augments the natural stretch caused by the kinesthetic device), (2) opposing tactile stimulation, and (3) natural tactile stimulation, respectively.

By adding a skin-stretch in two opposite directions, we aimed to distinguish between several different hypotheses. First, the additional stretch in the same direction as the force-field may increase the perceived load forces, and a stretch in the opposite direction may decrease the perceived forces (although the latter prediction is less certain as skin-stretch in the opposite direction was almost never studied [42]). This will result in greater manipulation forces and stronger adaptation of the movement path in the *same direction* group, and smaller manipulation forces and weaker adaptation of the movement path in the *opposite direction* group. If a similar internal representation is used to control grip forces, the effect on grip forces will be similar to the effect on the manipulation forces. Second, instead of creating the illusion of larger or smaller forces, artificial skin stretch can create the illusion of a more slippery contact. In this case, all the groups will have similar adaptation of movement path and manipulation forces, and only the two groups with additional stimulation will increase their grip forces compared to the group without additional stimulation. Third, the tactile stimulation may increase the uncertainty regarding the load forces rather than a bias in their size or in the mechanical properties of the contact. In this case, both groups with additional stimulation would decrease their adaptation of movement path and manipulation forces, and increase their grip forces regardless of stimulation direction.

## Methods

### Participants

Forty-five right-handed healthy volunteers participated in the experiment. Participants signed the informed consent form as approved by the Human Participants Research Committee of Ben-Gurion University of the Negev, Be’er-Sheva, Israel. The participants were all naive to the purpose of the experiment and were reimbursed for their participation.

### Experimental setup

**Fig. 1:**
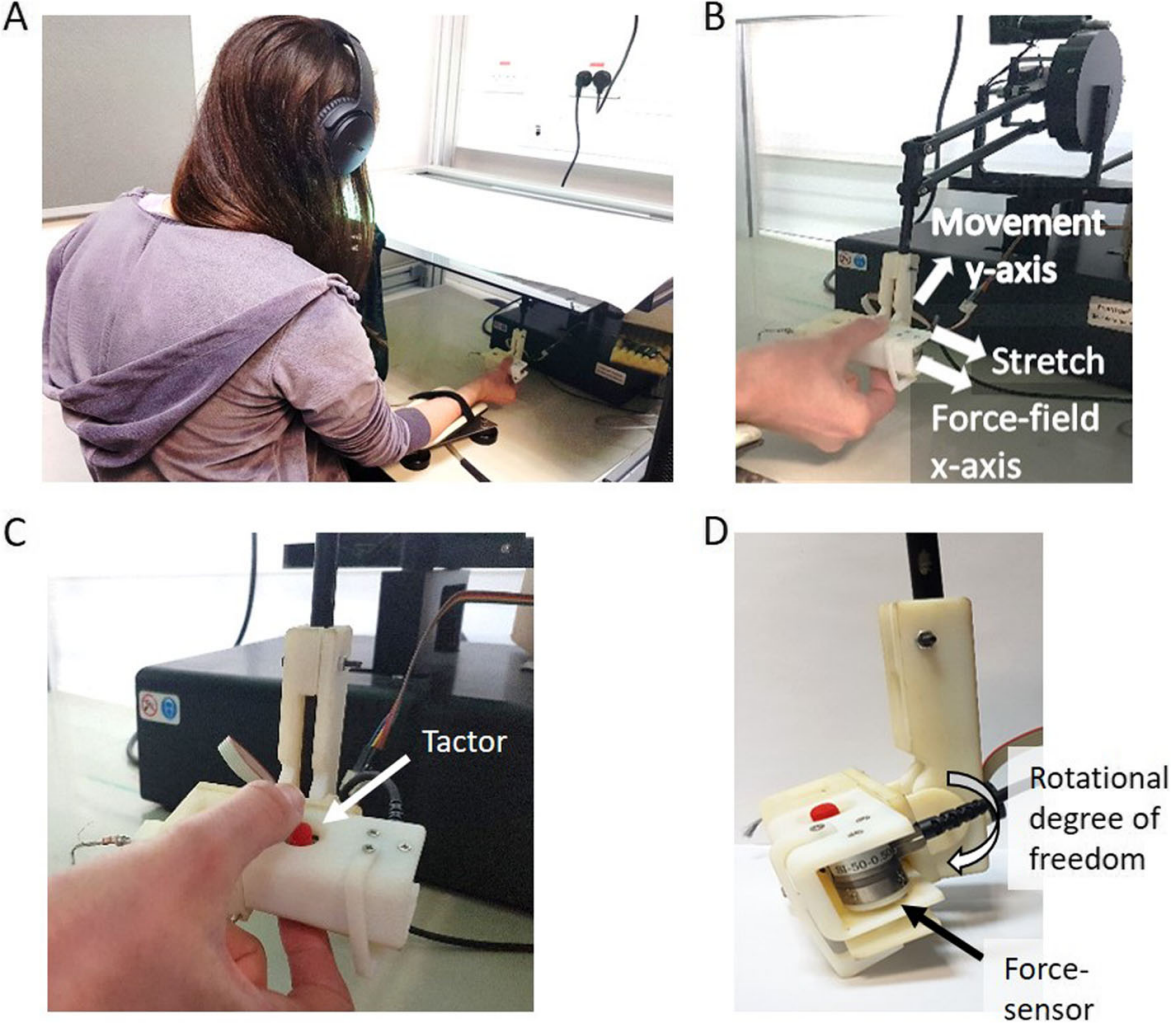
Experimental setup. **a** The participants were seated in front of a screen, while holding the skin-stretch device. Participants’ arm was attached to an air-sled wrist supporter, and they wore noise-cancelling headphones. **b** The skin-stretch device was attached to a haptic device that was used to apply the force-field and to record position, velocity and forces. Desired movement direction was in the frontal, *y*-axis, away from the body of the participant and in the horizontal plane, and the force-filed and skin-stretch were applied in lateral, *x*-axis. **c** Participants’ thumb and index finger were located on the moving tactors (red pins with high-friction surface) that stretched the skin of the finger pad. **d** A force sensor was used to record the grip force that was applied on the skin-stretch device. In addition, we added a rotational degree of freedom in the connection between the skin-stretch device and the haptic device, such that throughout the movement the stretch will be applied in a perpendicular direction to the desired movement direction

During the experiment, participants sat in front of a screen with their upper body covered by a sheet and performed reaching movements in a virtual reality environment using a six degrees of freedom robotic arm: PHANTOM® Premium™ 1.5 haptic device (Geomagic®) (Fig. 1a). On the robotic handle, we attached a custom-built skin-stretch device, and the participants held the haptic device with their thumb and index finger placed on the skin-stretch device in a precision grip (Fig. 1b). The movement of the robotic arm controlled a cursor that was displayed on the screen. We constrained participants’ movements to the horizontal plane and provided support against gravity by placing their forearm on an air sled wrist-supporter that reduced the friction with the surface. To eliminate auditory cues from the different devices, throughout the entire experiment, the participants wore noise-cancelling headphones (Bose QuietComfort 35 II).

### Skin-stretch device

Aiming to understand the integration between tactile and kinesthetic information during adaptation to a force perturbation, we built a 1 DOF skin-stretch device similar to the one that was used in [4], with several modifications to fit the needs of the current study. We modified the configuration of the device such that the skin-stretch will be applied in the horizontal plane and perpendicularly to the desired movement direction (Fig. 1a-b). The device consisted of two tactors (red pins with high-friction surface, a Lenovo TrackPoint caps, Fig. 1c), DC micro motor (Faulhaber, series 1516-SR) that was used to move the tactors in one dimension, a spur gearhead (Faulhaber, series 15/8 with gear ratio of 76:1), and an encoder (Faulhaber, series IE2–1024). The connection of the encoder to the computer was via a USB RS232 serial adapter.

To measure the grip force that was applied on the device, we had a force sensor (Nano17, ATI Industrial Automation, Fig. 1d) that was placed on the edge of the device. The grip force was transferred to the force sensor through a ‘door’ that was compressing the sensor in one side relatively to the amount of grip force that the participants applied on the other side. This structure allowed us to measure only a downscaled version of the grip force rather than the exact magnitude of the force between participants’ fingers, but this downscaling was consistent through the low of angular momentum conservation. The grip force that the participants applied on the tactors maintained friction, and as a result, the movement of the tactors caused skin-stretch.

The device was attached to a PHANTOM® Premium™ 1.5 haptic device (Geomagic®), and applied tangential skin deformation on the thumb and index finger such that only the tactile mechanoreceptors in the skin were stimulated (Fig. 1b). To make sure that the stretch will be applied in the horizontal plane, we added a degree of freedom in the connection between the skin-stretch device and the haptic device (Fig. 1d), such that the participants could maintain the skin-stretch device perpendicular to the surface of the movement.

### Protocol

**Fig. 2:**
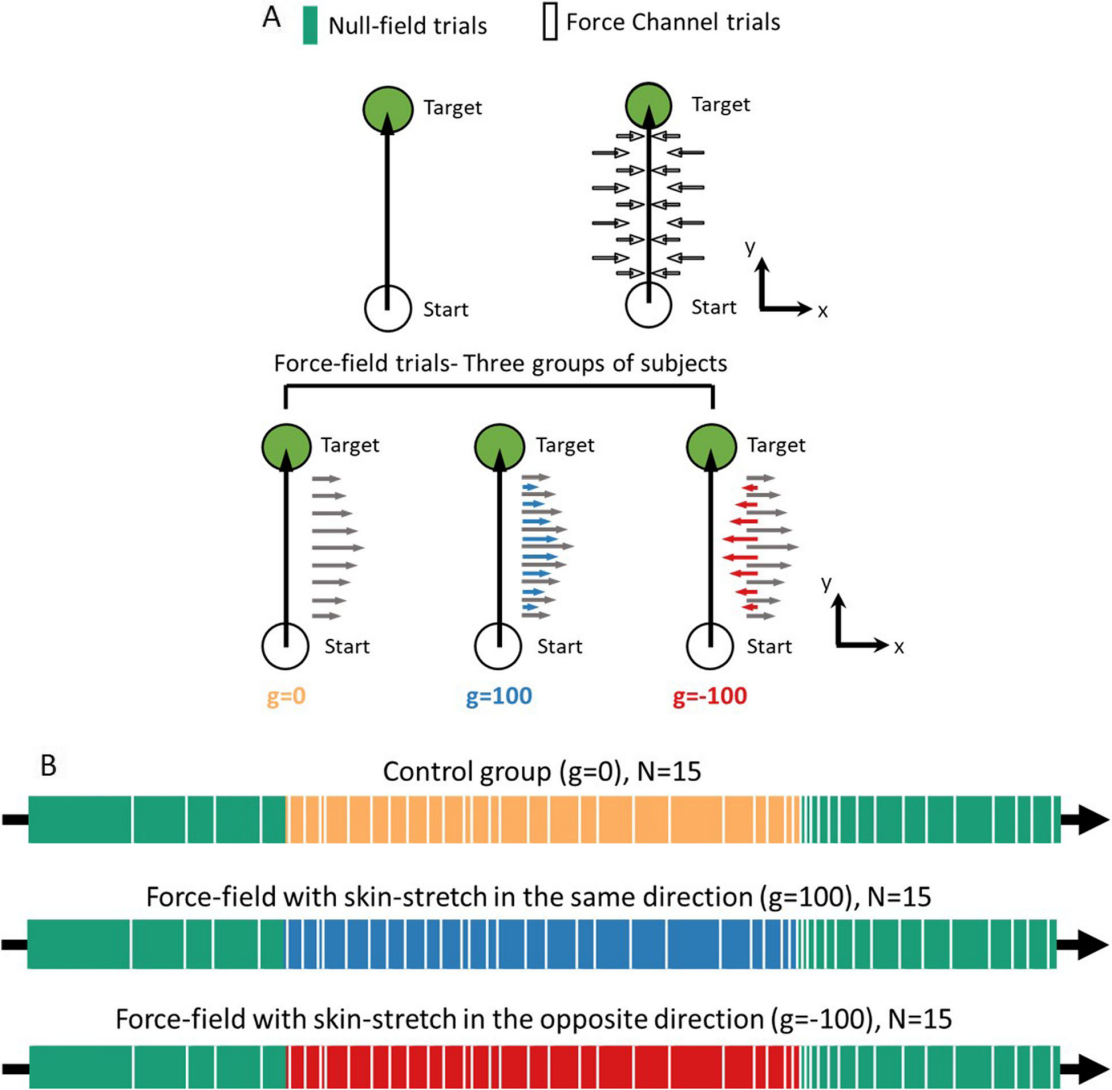
Experimental protocol. **a** In each trial, participants were required to make a *reaching movement*: move a cursor from a start position (white circle) toward a target (green circle). During null-field trials, no force-field was presented. In force channel trials, participants’ movement was constrained to straight trajectory by using virtual walls. In force-field trials, a velocity-dependent force was applied, perpendicular to movement direction from start to target. Here, we had three conditions: (1) g = 0 – control group (yellow) with only force-field, (2) g = 100 – force-field with skin-stretch in the same direction (blue), and (3) g = − 100 – force-field in one direction and skin-stretch in the opposite direction (red). **b** The experiment was divided into three sessions: Baseline (green bar), Adaptation (yellow/blue/red bar), and Washout (green bar). During the Baseline and Washout sessions, null-field trials were presented. During the Adaptation session, we presented force-field trials with and without augmented tactile information. Throughout the experiment, in randomly selected trial we applied force channel trials (white bar, see Methods for details)

The experiment was administered by a dedicated C++ code. Using the haptic devices, we applied a velocity-dependent kinesthetic and tactile stimulation in the lateral direction (*x*-axis) that was perpendicular to the desired frontal movement direction (*y*-axis, away from the body) (Fig. 1b). The force-field, designated from now as *load force* (LF), was applied by the Phantom haptic device such that:

$$ LF(t)=b\left[\begin{array}{cc}0& 1\\ {}0& 0\end{array}\right]\dot{X}(t) $$

where $$ LF(t)=\left[\begin{array}{c}L{F}_x(t)\\ {}L{F}_y(t)\end{array}\right] $$ is the applied force, $b=10\frac{N\bullet s}{m}$ is the velocity gain, and $$ \dot{X}(t)=\left[\begin{array}{c}\dot{x}(t)\\ {}\dot{y}(t)\end{array}\right] $$ is the velocity. Accordingly, the force in *x*-axis depended on the velocity in *y*-axis. In addition to the force-field, in two of the groups, we applied velocity-dependent skin-stretch, by means of a displacement of tactors that moved tangential to the skin of the thumb and the finger of the participants, in the same or in the opposite direction to the applied force. A third group did not receive any additional tactile stimulation (Fig. 2a). To apply the stretch, we controlled the location of the tactors such that:

$$ {x}_{tactor}=g\left[0\kern0.5em 1\right]\dot{X}(t) $$

where $g=\Big\{0\frac{mm\bullet s}{m},100\frac{mm\bullet s}{m},-100\frac{mm\bullet s}{m}$ } is the tactors’ displacement gain, and $$ \dot{X}(t)=\left[\begin{array}{c}\dot{x}(t)\\ {}\dot{y}(t)\end{array}\right] $$.

The experiment consistent of 400 trials in which the participants had to perform reaching movements from a start point toward a target. A trial was initiated when the participants located a yellow circular cursor (1.6 cm diameter) on the start point (white circle, 2 cm diameter) for a fixed amount of time. Then, the start point changed the color to green, instructing the participant to start a fast reaching movement toward a black circular target (2 cm diameter), located 10 cm away from the start position along the y-axis. A trial ended when the velocity was less than 0.05 cm/s. To guide the participants to move with a duration in a desired range, following the movement, we displayed a feedback based on the duration of the movement. When the duration was lower than 0.4 s the words “Move Slower” appeared on the screen, and when the duration was higher than 0.6 s the words “Move Faster” were displayed. In addition, if the participant passed the target, we provided a feedback of “Stop on the target”. When the duration of the movement was in the desired range and the participant stopped on the target, the word “Exact” was displayed. To motivate participants to perform accurate movements in the desired timing, we displayed a success rate that calculated the percentage of exact trials from all the trials that were performed.

The experiment consisted of three sessions: Baseline, Adaptation, and Washout (Fig. 2b). In the Baseline session (100 trials), the participants performed reaching movements without any perturbation. In the Adaptation session (200 trials), we exposed the participants to a velocity-dependent force-field with or without skin-stretch. The Washout session (100 trials) was similar to the Baseline: we abruptly removed the perturbation. During the experiment, we had 44 force channel trials – 4 trials in the Baseline session, 25 in the Adaptation, and 15 in the Washout session (Fig. 2a). In these trials, the participants performed the same reaching movements, and the Phantom haptic device applied virtual walls that constrained the movement to be a straight movement from start to target by using stiffness (500 N/m) and damping (2 Ns/m). None of the force channel trials include artificial skin stretch stimulation in any of the groups. These trials enabled us to estimate the *manipulation forces* (MF) that the participants applied to compensate for the perturbing force-field throughout adaptation by recording the forces that were applied by the channel, similarly to [16–18].

In this study, we divided the participants to three groups according to the exposure to skin deformation: (1) a *control group* without skin-stretch *g = 0* (*N* = 15), (2) skin-stretch in the *same direction* as the force-field with a gain of *g = 100* [mm*s/m] (N = 15), and (3) skin-stretch with gain of *g = − 100* [mm_•_s/m] such that the stretch is in *opposite direction* to the applied force-field (*N* = 15).

### Data analysis

Using the haptic device, we recorded the position, velocity and applied forces. In addition, we recorded the grip-forces using a force sensor. All data were recorded at 80 Hz and analyzed off-line via a custom-written MATLAB code (The MathWorks, Inc., Natick, MA, USA). All the signals were filtered with a low pass zero phase Butterworth filter with a cutoff frequency of 10 Hz (MATLAB function filtfilt()). To match the length of the signals, we normalized the time of each signal between [0 1], and interpolated each signal to have the same number of samples (MATLAB function interp1()).

To quantify the effect of the augmented tactile information on the kinematics of reach movements we calculated the *position error* in each trial. This was calculated as:

1 $$ position\ error=\max \left(x(t)\right) $$

where *x*(*t*) is the position signal in x-axis.

To quantify the contribution of the augmented tactile information to the internal representation that is used to control manipulation forces, we estimated the manipulation forces during force channel trials. To assess the similarity between the manipulation (*MF*) and load forces (*LF*), we calculated the *adaptation percentage*, by computing the regression between the manipulation force in a force channel trial (trial *n*) and the load force in a trial before (trial *n-1*) [16, 49–52].

2 $$ MF={b}_1\bullet LF+{b}_0 $$

3 $$ Adaptation\ percentage={b}_1\bullet 100\% $$

where *b*_1_ is the regression coefficient and *b*_0_ is the offset. To fully compensate for the load forces, the participants had to apply manipulation forces that are similar to the load forces. Therefore, we expect the adaptation measure to increase as the participants develop an internal representation of the perturbation. We also expected that the augmented tactile information might contribute to building the internal representation faster and reach higher level of adaptation.

We followed [13–15] and assumed that the internal representation is formed using position and velocity primitives. To quantify the effect of the augmented tactile information on the primitives that are used to plan the manipulation forces, we calculated a regression between the manipulation forces and the position and velocity state variables. Because the manipulation forces that are applied during force channel *n* reflect the expectation of the force-field based on preceding trials, the representation analysis was performed by fitting a model for the manipulation force in a force-channel *n*, by using state information of position and velocity from trial *n-1* such that

4 $$ M{F}_{fitted}=k\bullet {q}_p\bullet y(t)+b\bullet {q}_v\bullet \dot{y}(t) $$

where *y*(*t*) and $$ \dot{y}(t) $$ are the position and velocity in the desired movement direction. *k* and *b* are the normalized gains of the *position and velocity primitives*. To match the units of the two primitive signals to force units, we used the normalization factors *q*_*p*_ and *q*_*v*_. These factors were selected such that the peak perturbation forces will be equal between force-fields that depends only on one state variable [13, 16]. The velocity normalization gain was chosen as the velocity gain of the force-field $$ {q}_v=10\frac{N\bullet s}{m} $$ for all groups. For the position normalization gain we calculated the mean maximum forces that were applied during force-field trials across all participants *f*_max_, and divided it by the maximum displacement *p*_max_ = 10*cm*. Therefore, the position normalization gain for each group was: g = − 100: $$ {q}_p=0.44\ \raisebox{1ex}{$N$}\!\left/ \!\raisebox{-1ex}{$ cm$}\right. $$, g = 0: $$ {q}_p=0.42\ \raisebox{1ex}{$N$}\!\left/ \!\raisebox{-1ex}{$ cm$}\right. $$, and g = 100: $$ {q}_p=0.45\ \raisebox{1ex}{$N$}\!\left/ \!\raisebox{-1ex}{$ cm$}\right. $$. This entire analysis was conducted as in [16].

The effect of the perturbation on the applied grip forces was examined by measuring the *peak forces ratio*, i.e. the ratio between the maximum grip force (*GF*) to the maximum load force (*LF*). In a force-field trial, both signals were taken from the same trial. In a force channel trial, the grip force was taken from a force channel trial *n* and the load force was taken from a trial *n-1*.

5 $$ Peak\ Ratio=\frac{\max (GF)}{\max (LF)}. $$

This measure is an indication of the amount of grip force per amount of load force, and is expected to decrease as the internal representation is formed.

The peak forces ratio measure provides information about the strength of the grip, but it does not differentiate between different components of grip force control. A typical grip force trajectory is composed of a baseline grip force that is applied even when no load force is applied, and a modulation grip force that can be composed of a predictive component and a reactive component. Thus, for each force-field and force channel trial in Adaptation we evaluated the *baseline grip force* as *GF*(*t* = 0), and the *modulation grip force ratio* as:

6 $$ modulation\ ratio=\frac{\Delta  GF}{\Delta  LF}=\frac{\max (GF)- GF\left(t=0\right)}{\max (LF)- LF\left(t=0\right)}. $$

In force channel trials, no net force is applied at the contact with the fingers of the participants, and therefore, the modulation grip force ratio quantifies the predictive component. In regular trials with force-field, the modulation grip force ratio includes both the predictive but also the reactive components.

### Statistical analysis

Statistical analyses were performed using a custom-written Matlab code (The MathWorks, Inc., Natick, MA, USA). Throughout the manuscript all our statistical model included a between participants factor of skin-stretch group, and a within participant factor that was specific to each analysis. Therefore, for statistical analysis we used a 2-way mixed model ANOVA with between factor of *group* (g = − 100/g = 0/g = 100), and the tested within factor. For the individual multiple comparisons, in the *position error* analysis, we were interested in the differences between the different stages of the protocol (the beginning of the Adaptation session, the end of the Adaptation session, and the beginning of the Washout session) and the end of the Baseline session. In addition, to ensure that there was a significant reduction of position error throughout Adaptation, we examined the difference between early to late Adaptation. For the rest of the analyses, we performed post-hoc comparisons. Significant effects were defined as those with probability level of *p* < 0.05. When significant effects were found, a post-hoc t-test was conducted with Holm’s correction for multiple comparisons.

## Results

### An artificial stretch of the skin does not affect the movement paths

**Fig. 3:**
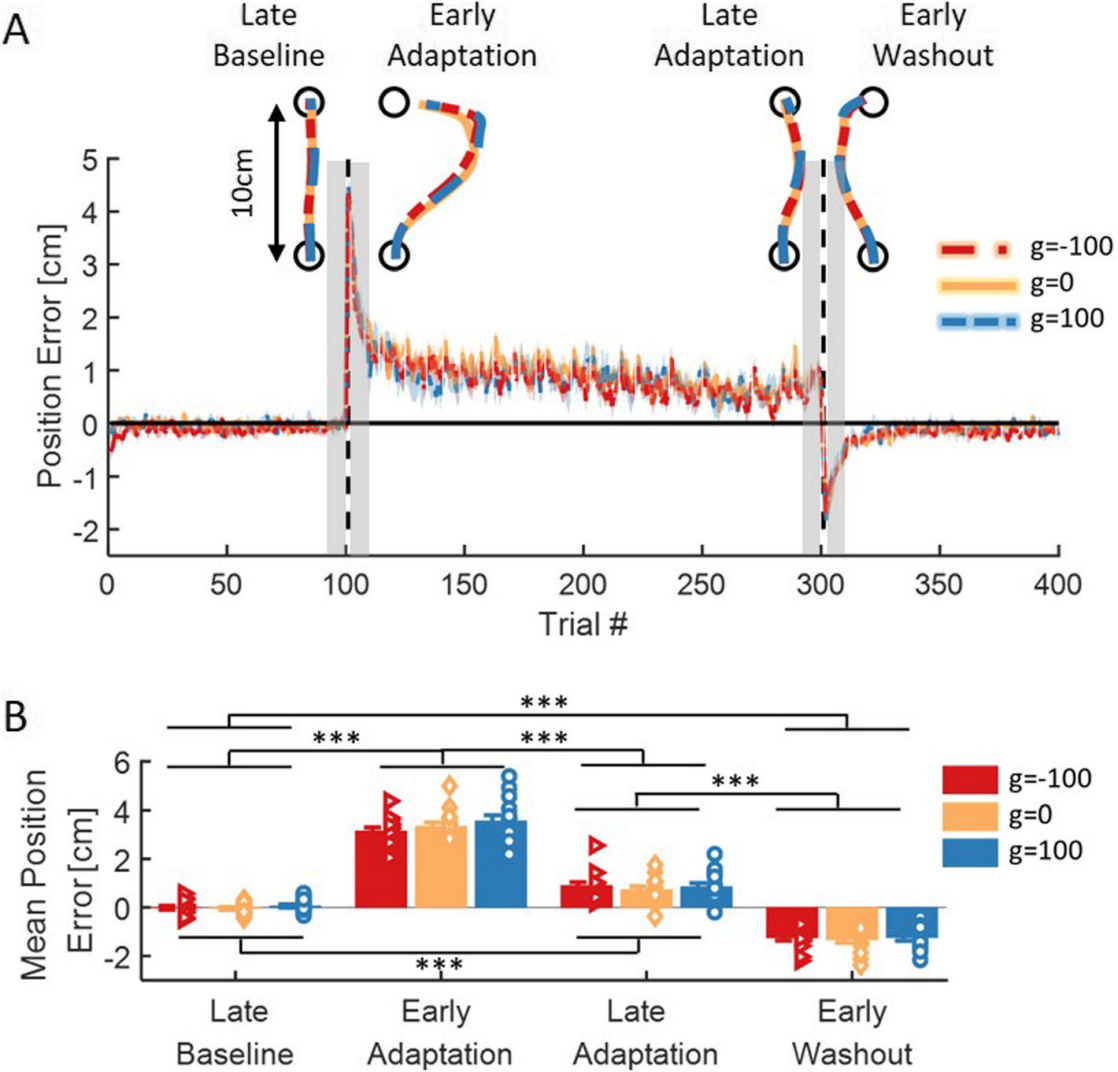
Position error - maximum deviation in the axis perpendicular to the desired movement direction (*x*-axis). **a** Mean position error and SE (shaded region) for the three groups of g = − 100 (red), g = 0 (yellow), and g = 100 (blue). Dashed black lines represents the different sessions of Baseline, Adaptation and Washout. For each stage in the experiment (Late Baseline- LB, Early Adaptation- EA, Late Adaptation- LA, Early Washout- EW), a typical trajectory is presented. Shaded gray regions indicate the trials that were used for the statistical analysis. **b** Mean positional error over three movements in each stage of LB, EA, LA, and EW. Colors are as in (**a**). Error bars represent ±SE, and the dots represent the data from each participant. ***p < 0.001

To probe for the effect of the skin-stretch on adaptation, we first examined the *position error*, which was defined as the maximum lateral deviation from a straight path (Eq. 1). We saw that when the force-field was first applied, regardless to the direction and magnitude of the skin-stretch, all participants deviated in the same direction of the force-field. With continued exposure, participants reduced the positional error, although adaptation was not fully accomplished. In addition, when the perturbation was abruptly removed, the participants deviated to the opposite direction. Importantly, there was no difference between the different experimental groups, as evident in the example trajectories as well as in the averaged adaptation curves (Fig. 3a).

To quantify adaptation, we compared the positional errors between four stages of exposure to the perturbation: end of the Baseline session (Late Baseline- LB), beginning of the Adaptation session (Early Adaptation- EA), end of the Adaptation session (Late Adaptation- LA), and the beginning of the Washout session (Early Washout- EW). We performed a 2-way mixed model ANOVA with one between participants factor of *group* (g = − 100/g = 0/g = 100), and one within participants factor of *stage* (LB/EA/LA/EW). The statistical analysis revealed a significant main effect of *stage* (*F*_3,126_ = 632.38, *p* = 9.65e-76), and no main effect of *group* or interaction between *group* and *stage* (*F*_2,42_ = 1.08, *p* = 0.34 and *F*_6,126_ = 0.65, *p* = 0.68, respectively). Following a planned comparisons analysis, we found that for all groups there was a significant deviation when the perturbation was first applied compared to the end of the Baseline (g = − 100: t_42_ = 16.35, *p* = 4.75e-19, g = 0: t_42_ = 17.5, *p* = 3.97e-20, g = 100: t_42_ = 18.13, *p* = 1.05e-20). This deviation was reduced as adaptation progressed (difference between early and late Adaptation: g = − 100: t_42_ = 10.5, *p* = 1.53e-12, g = 0: t_42_ = 12.16, *p* = 1.41e-14, g = 100: t_42_ = 12.67, *p* = 3.69e-15), although the participants did not gain Baseline performances at the end of Adaptation (g = − 100: t_42_ = 5.88, *p* = 3.48e-6, g = 0: t_42_ = 5.01, *p* = 6.28e-5, g = 100: t_42_ = 5.11, *p* = 4.53e-5). When the perturbation was abruptly removed, the participants deviated to the opposite side compared to the Baseline (g = − 100: t_42_ = 8.63, *p* = 4.56e-10, g = 0: t_42_ = 9, *p* = 1.42e-10, g = 100: t_42_ = 9.11, *p* = 9.98e-11, Fig. 3b). This shows that the participants adapted to the perturbation by modifying their movements and exhibited aftereffects of opposite deviation when the perturbation was removed. However, the analysis revealed no difference between the three groups, which confirms that the additional skin-stretch in either of the directions did not affect kinematics.

### The group with a skin-stretch in the opposite direction adapted the manipulation force more than the group with a skin-stretch in the same direction as the applied force

**Fig. 4:**
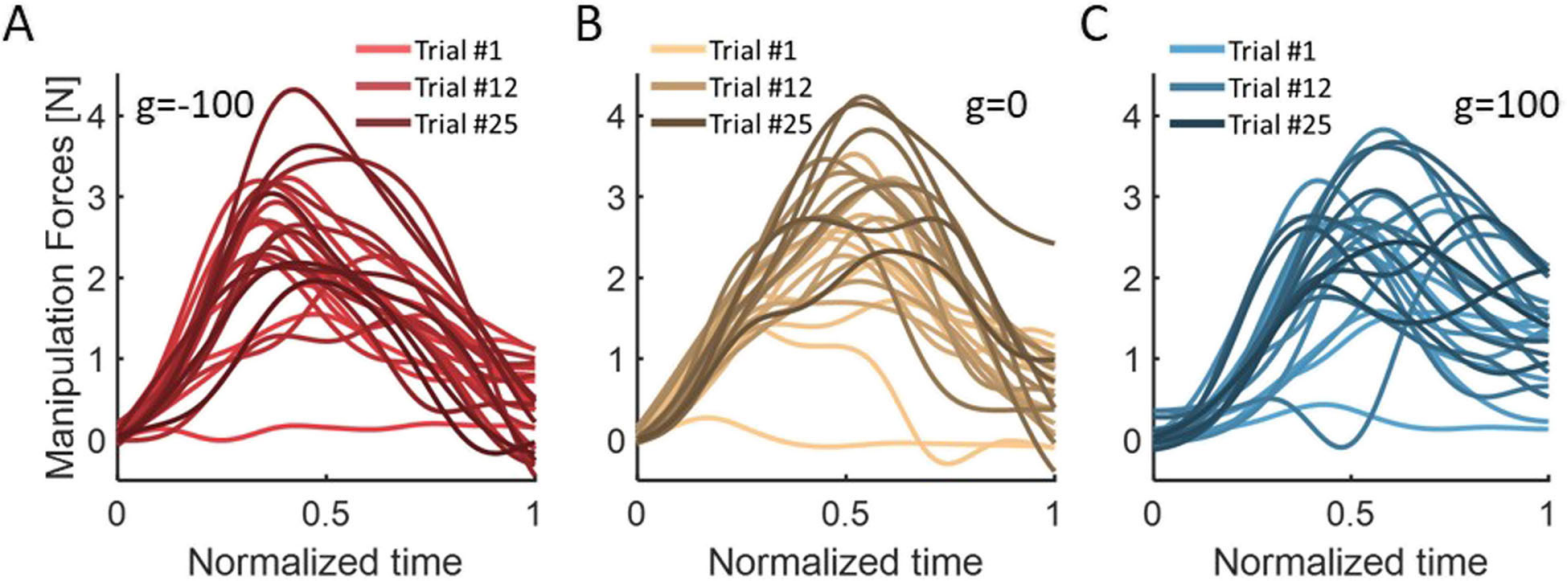
Manipulation forces from all force channel trials in the Adaptation session from a typical participant in each group of **a** g = − 100, **b** g = 0, and **c** g = 100. Colors are changing from light to dark as adaptation progresses

To probe the effect of the applied skin-stretch on the internal representation that is used to generate manipulation forces, we examined the manipulation forces that the participants applied during force-channel trials, and compared them with the load forces that the participants experienced. First, we looked at the manipulation forces from all force-channel trials in the Adaptation session. The results of a representative participant from each group are presented in Fig. 4. We saw that as adaptation progressed, the manipulation forces became larger and more similar to the bell-shaped load forces.

**Fig. 5:**
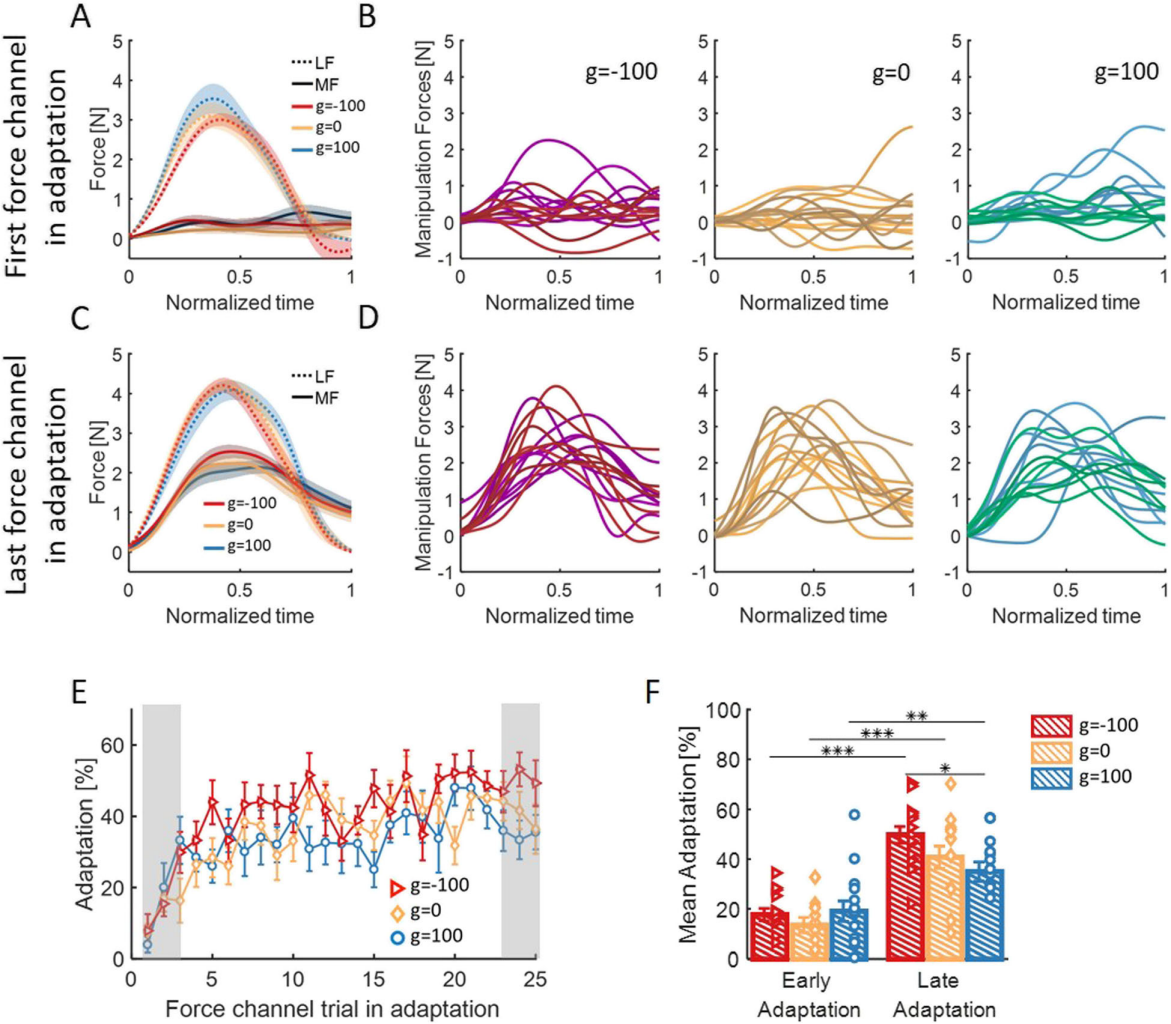
The effect of adaptation on the manipulation forces. **a** Mean signals of the manipulation forces (MF, solid line) applied in the first force channel in the adaptation session, and the load forces (LF, dashed line) from the previous trial, for the three groups of g = − 100 (red), g = 0 (yellow), and g = 100 (blue). Shaded regions represent ±SE. **b** Manipulation forces for each participant in the first force channel in Adaptation. The signals are presented for each group separately, from left to right: g = − 100, g = 0, and g = 100. **c** and **d** are similar to (**a**) and (**b**) for the last force channel in the adaptation session. **e** Adaptation percentage measured by the regression coefficient between the manipulation forces in a force channel trial and the load forces from the preceding trial. Colors are as in (**a**), and error bars represents ±SE. Shaded gray regions indicate the trials that were used for the statistical analysis. **f** Mean and ± SE of adaptation percentage in the two stages of Adaptation: Early – 3 first force channel trials in Adaptation, and Late – 3 last force channels in Adaptation. Colors are as in (**a**), and the dots represents the data from each participant. *p < 0.05, **p < 0.01, ***p < 0.001

In addition, we compared between the manipulation forces that were applied during either the first or the last force-channel trials (trial *n*) and the load forces that were applied a trial before (trial *n-1*, Fig. 5b and d). We then averaged the two signals across participants (Fig. 5a and c for the beginning and end of Adaptation, respectively). We saw that for all groups, the participants adjusted their manipulation forces throughout adaptation, such that at the end of the Adaptation session the manipulation forces were more similar to the load forces then in the beginning of the Adaptation session.

To quantify the similarity between the manipulation and load forces, we used a linear regression between the signals, and calculated the *adaptation percentage* (Eq. 2 and Eq. 3). For all groups, the *adaptation percentage* increased with continued exposure to the perturbation (Fig. 5e). To assess the increase and the difference between the three groups, we examined the adaptation of each group in the beginning (first 3 force channel trials) and end (last 3 force channel trials) of the Adaptation session. We used a 2-way mixed model ANOVA with one between factor of *group* and one within factor of *stage* in the Adaptation session (early/late). We found a significant main effect of *stage* and a significant interaction between *group* and *stage* (*F*_1,42_ = 88.38, *p* = 6.85e-12 and *F*_2,42_ = 3.22, *p* = 0.04, respectively) and no main effect of *group* (*F*_2,42_ = 1.91, *p* = 0.15). At the end of adaptation, the group with opposite skin-stretch had higher percent of adaptation than the group with skin-stretch in the same direction as the applied force-field (t_42_ = 2.66, *p* = 0.03, Fig. 5f). This indicates that applying a skin-stretch in the opposite direction caused participants to develop a better representation of the applied force-field.

**Fig. 6:**
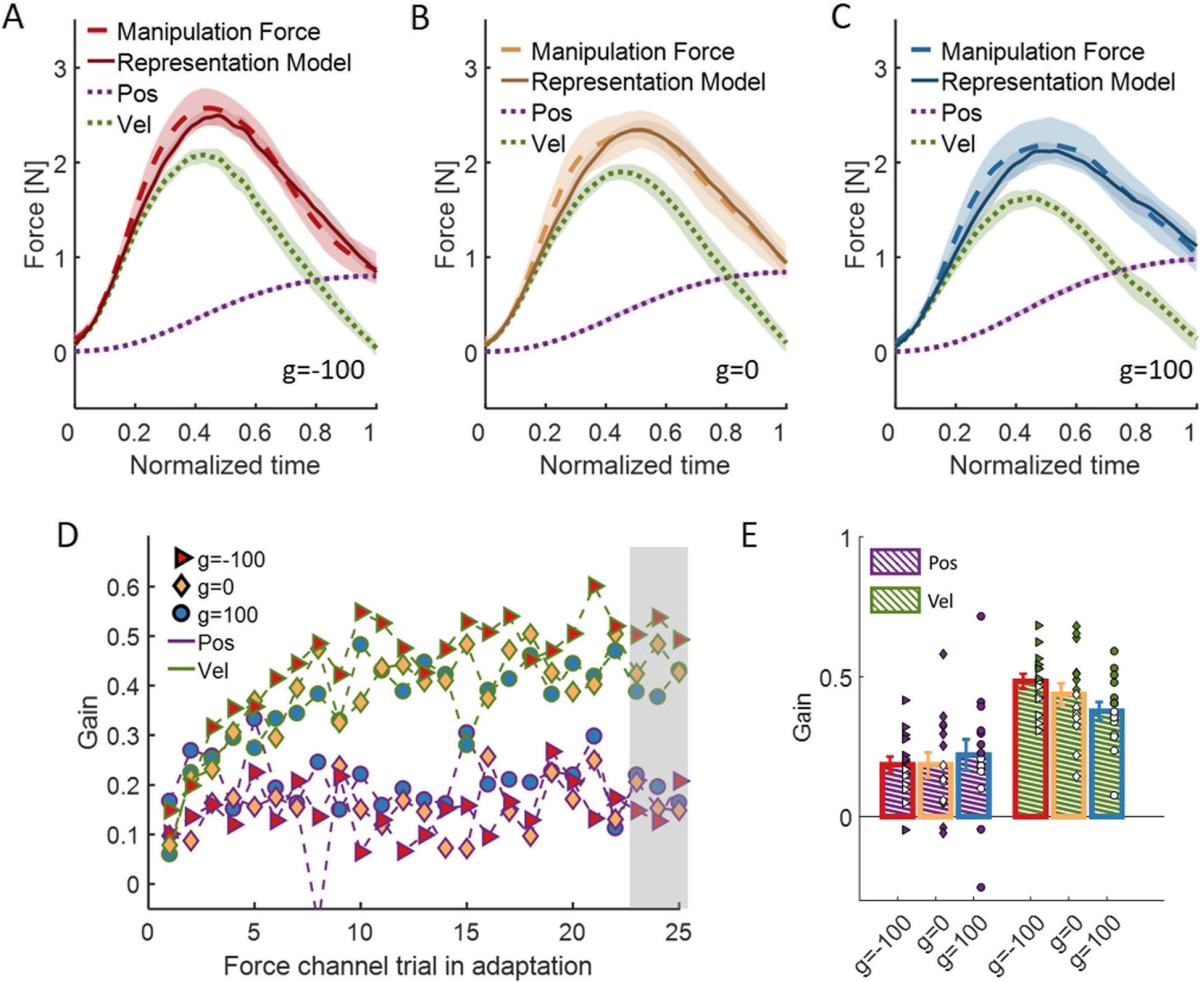
Representation analysis. **a** The actual manipulation forces (dashed red) and model (solid red) for the group with skin-stretch in the opposite direction to the force-field (g = − 100). The motor primitives that were used for modeling the manipulation force are position (dashed purple), and velocity (dashed green). **b** and **c** are as in (**a**) for the control group (g = 0, yellow) and the group with skin-stretch in the same direction as the force-field (g = 100, blue), respectively. **d** The mean gain across participants of the position (purple) and velocity (green) primitive that was required in order to model the manipulation forces in every force channel trial in Adaptation. The results are presented for the three group of g = − 100 (red triangle) and g = 0 (yellow diamond), and g = 100 (blue circle). Shaded gray region indicates the trials that were used for the representation and statistical analysis. **e** Mean and ± SE over the three last force channel trials in Adaptation for each motor primitive in every group. Colors are as in (**d**), and the dots represents the data from each participant

To understand better the difference in the internal representation between the groups, we analyzed the effect of the additional skin-stretch on the motor primitives that are used for representation of the force-field for each group. It was proposed that *position and velocity primitives* are used to represent velocity-dependent force-field perturbations [13–15]. We followed [13], and calculated a regression between the manipulation force that the participants applied during a force channel trial to the state information from the preceding trial (Eq. 4, Fig. 6a-c). From the development of the primitives with continued exposure to the perturbation, we can see that throughout most of the adaptation, the group with opposite skin-stretch used more the velocity component and less the position component than the group with skin-stretch in the same direction (Fig. 6d). To quantify these results, we fitted a 2-way mixed model ANOVA with between-participants factor of *group* and within-participants factor of *motor-primitive* (position/velocity). For this representation analysis, we used the three last force channel trials in the Adaptation session. We found no significant effect of *group* and no interaction between *group* and *motor-primitive* (*F*_2,42_ = 0.88, *p* = 0.42 and *F*_2,42_ = 1.13, *p* = 0.33, respectively) but significant main effect of *motor-primitive* (*F*_1,42_ = 35.62, *p* = 4.4e-7, Fig. 6e). This indicates that in all the three groups the velocity motor primitive contributes more than the position motor primitive to the representation of the viscous force-field for the control of manipulation forces, as expected. Interestingly, the difference in the weight of the velocity or position motor primitives alone cannot fully account for the difference in adaptation percentage between the two groups of skin-stretch in different directions.

### The group with skin-stretch in the same direction applied more grip force per amount of load force

**Fig. 7:**
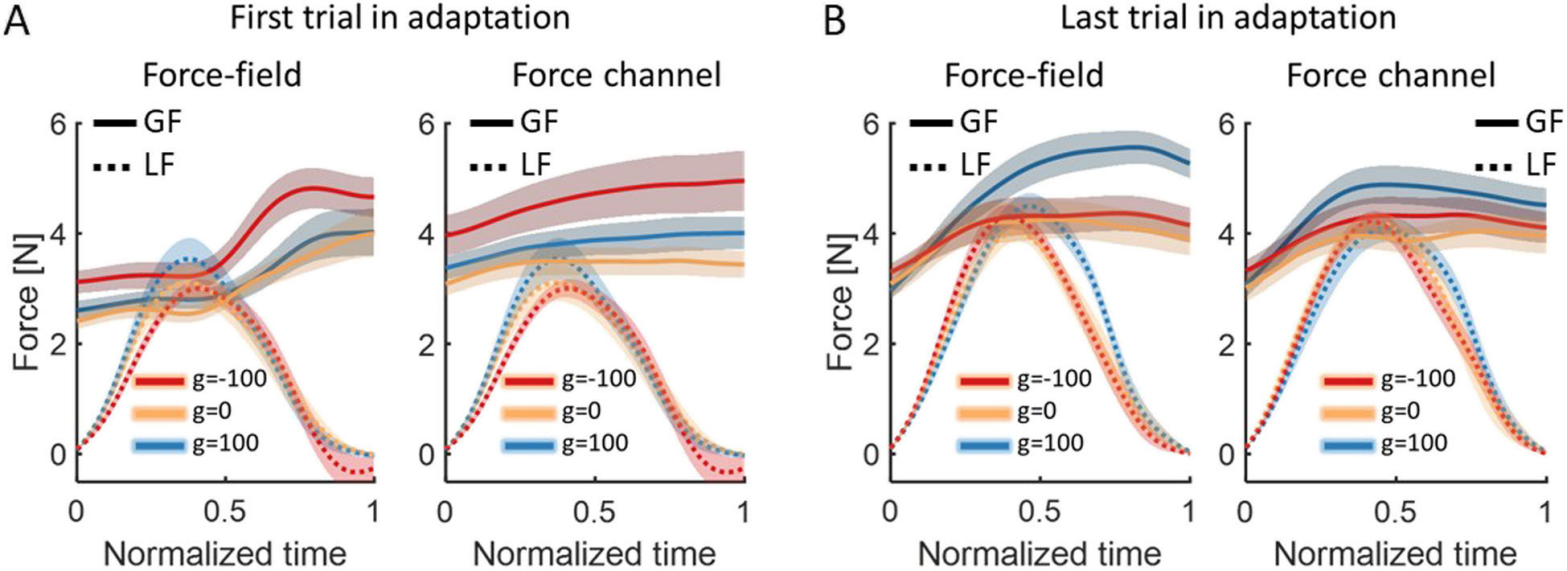
The effect of adaptation on the grip forces. **a** Mean signals across participants of the grip forces (GF, solid line) and the load forces (LF, dashed line) from the first force-field trial (left) and the first force channel trial (right) in Adaptation, for the three groups of g = − 100 (red), g = 0 (yellow), and g = 100 (blue). Shaded regions represent ±SE. **b** Same as (**a**) for the last force-field trial (left) and the last force channel trial (right) in Adaptation

To assess the effect of the perturbation on the control of grip force, we examined the grip force that the participants applied with respect to the load force that were applied on the participants. We saw that in the beginning of adaptation (Fig. 7a), the grip-force of the group with opposite skin-stretch was higher than of the other two groups, in both force-field (left panel) and force channel (right panel) trials. However, by the end of adaptation (Fig. 7b), the grip force of the group with skin-stretch in the same direction was much higher than the other two groups, even though the load forces were mostly similar in their amplitude (mean ± SE. Force-field trial (right panel): g = − 100: 4.3 ± 0.22, g = 0: 4.02 ± 0.18, g = 100: 4.5 ± 0.23. Force channel trial (left panel): g = − 100: 4.21 ± 0.18, g = 0: 4.2 ± 0.16, g = 100: 4.09 ± 0.25). To quantify this effect, we calculated the maximum grip force in each trial, and divided it by the maximum load force (*peak forces ratio*, Eq. 5). We repeated this analysis separately for regular force-field trials, in which the grip force includes both predictive and reactive components to the force and the skin-stretch, and in force channel trials, in which no net force or stretch is applied on the participants, and therefore, the grip force includes only predictive components.

**Fig. 8:**
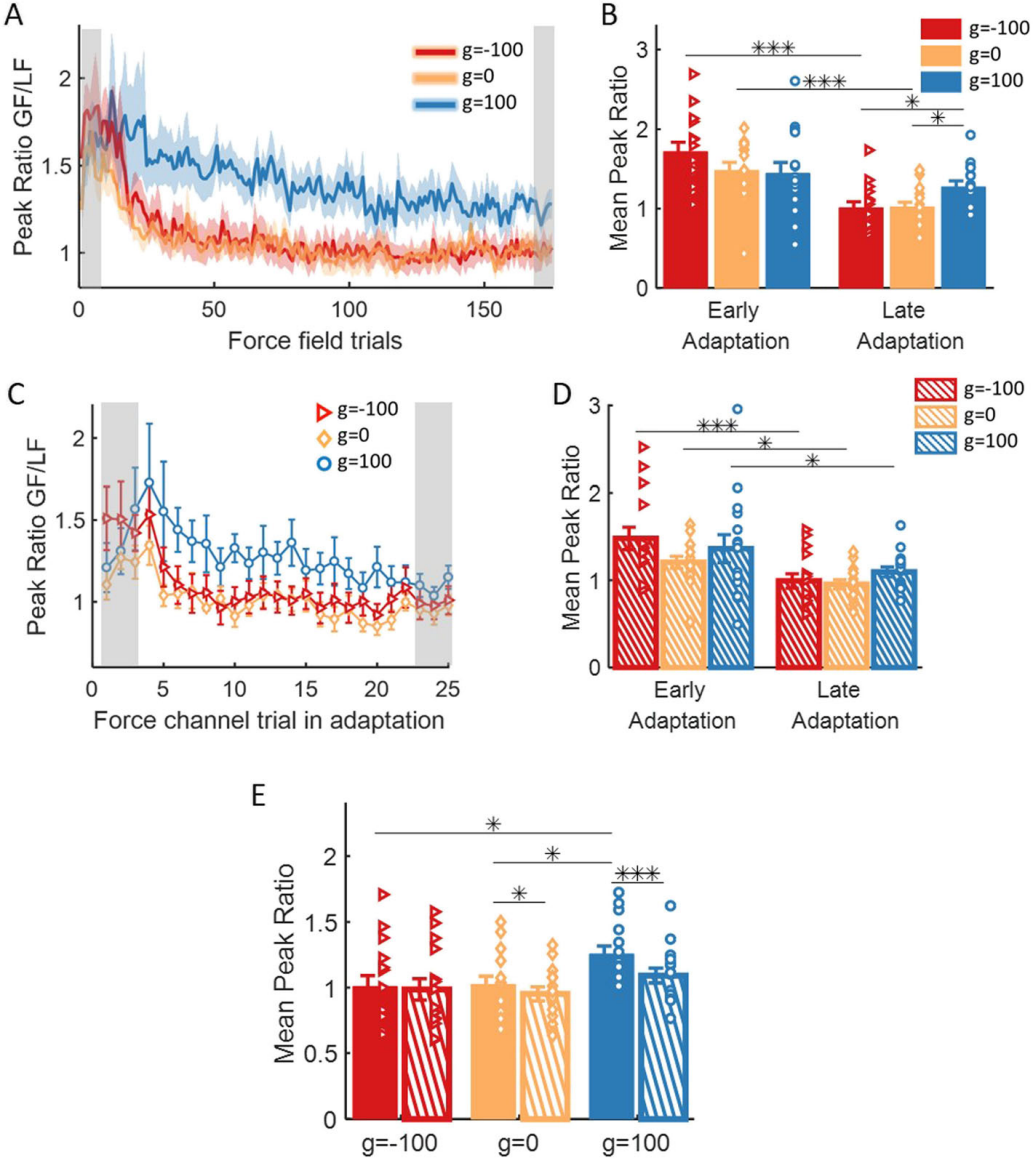
The effect of adaptation on the ratio between maximum grip force and maximum load force. **a** Mean and ± SE peak ratio across participants in all force-field trials for the three groups of g = − 100 (red), g = 0 (yellow), and g = 100 (blue). Shaded gray regions indicate the trials that were used for the statistical analysis. **b** Mean and ± SE of peak ratio measure in the two stages of Adaptation: Early – 3 first force-field trials, and Late – 3 last force-field trials. The dots represents the data from each participant. *p < 0.05, **p < 0.01, ***p < 0.001. **c** and **d** are as in (**a**) and (**b**) for all force channel trials in Adaptation. **e** Mean and ± SE of the last three force channel trials (dashed bar) and all force-field trials that were performed between these force channel trials (solid bar). Colors are as in (**a**), and the dots represents the data from each participant. *p < 0.05, **p < 0.01, ***p < 0.001

The results of the analysis of the force-field trials are depicted in Fig. 8a. In general, the group with skin-stretch in the same direction of the force-field applied more grip force per amount of load force than the two groups of opposite skin-stretch and control group. More specifically, both the opposite skin-stretch group and the control group decreased the peak ratio with adaptation; i.e., in these groups, the participants applied less grip force per amount of load force as the internal representation was formed. The decrease of the peak ratio for the group with skin-stretch in the same direction was much smaller. To support these observations, we fitted a 2-way mixed model ANOVA with between factor of *group* and within factor of *stage* in Adaptation (early/late). The analysis yielded a significant effect of *stage* and interaction between *group* and *stage* (*F*_1,42_ = 42.43, *p* = 7.22e-8 and *F*_2,42_ = 5.11, *p* = 0.01, respectively), but no significant effect of *group* (*F*_2,42_ = 0.65, *p* = 0.52). A post-hoc t-test showed that both groups of opposite skin-stretch and control, but not same direction group, significantly decreased the amount of grip force per amount of load force from beginning to end of Adaptation (g = − 100: t_42_ = 5.95, *p* = 4.62e-7, g = 0: t_42_ = 3.89, *p* = 3.5e-4, but g = 100: t_42_ = 1.44, *p* = 0.16). In the end of the Adaptation session, the group with skin-stretch in the same direction applied significantly more grip force per amount of load force than the group with opposite skin-stretch (t_42_ = 2.59, *p* = 0.03) and the control group (t_42_ = 2.51, *p* = 0.04, Fig. 8b). This shows that the augmented tactile information in the same direction as the force-field caused participants to apply more grip force per amount of load force, and impeded the reduction of grip force with adaptation.

The results of the analysis of the force channel trials are depicted in Fig. 8c. Similarly to the force-field trials, throughout adaptation, the group with skin-stretch in the same direction of the force-field applied more grip force than the other two groups. However, all the groups decreased the predictive grip force per amount of load from the beginning to the end of Adaptation. To quantify this observation, we fitted a 2-way mixed model ANOVA with one between factor of *group*, and one within factor of *stage* in the Adaptation (early/late). The analysis yielded a significant main effect of *stage* (*F*_1,42_ = 23.16, *p* = 1.95e-5), and no main effect of *group* or interaction between *group* and *stage* (*F*_2,42_ = 1.22, *p* = 0.31 and *F*_2,42_ = 1.18, *p* = 0.32, respectively). From the main effect of stage, we found a significant decrease in the peak ratio between early and late Adaptation for all groups (g = − 100: t_42_ = 4.03, *p* = 2e-4, g = 0: t_42_ = 2.1, *p* = 0.04, g = 100: t_42_ = 2.21, *p* = 0.03, Fig. 8d).

To understand the difference between the results observed in force channel and force-field trials, we wished to quantify directly the difference between the different components of predictive and reactive grip force. First, we compared directly between the measured peak ratio in force channel and force-field trials for all groups at the end of Adaptation. In order to verify that the data we used for the analysis is taken from the same phase of the adaptation process, we compared the last three force channel trials to all force-field trials that were performed between these force channel trials. We fitted a 2-way mixed model ANOVA with between factor of *group* and within factor of *trial* (force-field/force channel). The analysis yielded a significant main effect of *trial* and interaction between *group* and *trial* (*F*_1,42_ = 23.13, *p* = 1.97e-5 and *F*_2,42_ = 5.36, *p* = 0.008, respectively), and no main effect of *group* (*F*_2,42_ = 2.54, *p* = 0.09). Post-hoc analysis revealed a significant positive difference between force-field and force channel trials for both groups of skin-stretch in the same direction as the force and the control group (g = − 100: t_42_ = 0.72, *p* = 0.47, g = 0: t_42_ = 2.23, *p* = 0.02, g = 100: t_42_ = 5.28, *p* = 4.2e-6, Fig. 8e). Moreover, a significant difference between the groups was only observed in the force-field trials – the peak ratio of the group with skin-stretch in the same direction as the force was larger than the group with opposite skin-stretch (t_42_ = 2.51, *p* = 0.04) and the control group (t_42_ = 2.34, *p* = 0.04). This shows that the force-field causes an increase in the grip force compared to force channel trials. The skin-stretch caused an increase of this difference when applied in the same direction to the force-field, and canceled this effect when applied in the opposite direction to the force-field.

**Fig. 9:**
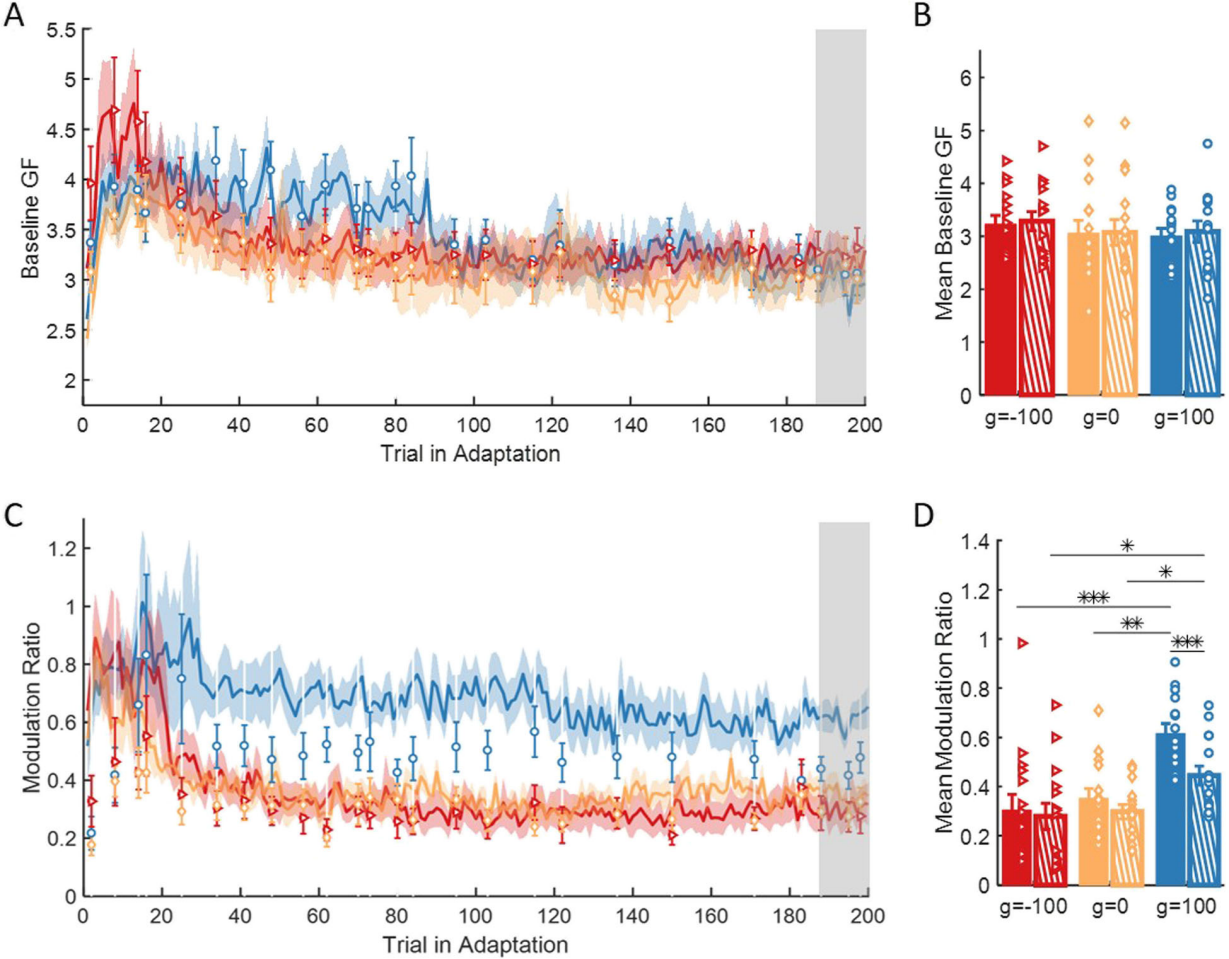
The effect of adaptation on the baseline grip force and the modulation between grip force and load force. **a** Mean and ± SE across participants of baseline grip force for the three groups of g = − 100 (red), g = 0 (yellow), and g = 100 (blue). Solid lines and dashed regions are for force-field trials, and markers and error bars are for force channel trials. Shaded gray regions indicate the trials that were used for the statistical analysis. **b** Mean and ± SE of the last three force channel trials (dashed bar) and all force-field trials that were performed between these force channel trials (solid bar). Colors are as in (**a**), and the dots represents the data from each participant. *p < 0.05, **p < 0.01, ***p < 0.001. **c** and **d** are as in (**a**) and (**b**) for the modulation between grip force and load force

However, the measure of peak ratio between grip force and load force does not differentiate between an increase of the predictive baseline grip force and the modulation of the grip force with the load force that can include both predictive and reactive components. Therefore, we repeated the last analysis but separately for the baseline grip force and the modulation of the grip force with load force. It is important to note that the modulation measurement and the peak ratio measurement are dependent: the former is calculated by reducing the baseline from the latter. First, we investigated the predictive *baseline grip force*, by examining the applied grip force in the beginning of the trial (t = 0) for each group, when no load force is applied (Fig. 9a-b). We found no difference between and within the groups, implying that the effect of the skin-stretch on the grip force is not a result of increasing the grip force by a fixed amount throughout the trial.

Then, we examined the *modulation ratio* between the grip force and the load force (Eq. 6). The results (Fig. 9c) showed a higher modulation for the group with skin-stretch in the same direction of the force, in both force-field and force channel trials. These observations were supported by the statistical analysis that was performed on data from the end of Adaptation. We fitted a 2-way mixed model ANOVA with between factor of *group* and within factor of *trial* (force-field/force channel). The analysis revealed a significant main effect of *trial*, *group* and interaction between *group* and *trial* (*F*_1,42_ = 22.16, *p* = 2.73e-5, *F*_2,42_ = 9.18, *p* = 5e-4 and *F*_2,42_ = 6.21, *p* = 0.004, respectively). For the group with skin-stretch in the same direction as the force, we found a significant increase in the modulation between force-field and force channel trials (g = − 100: t_42_ = 0.84, *p* = 0.4, g = 0: t_42_ = 1.76, *p* = 0.08, g = 100: t_42_ = 5.54, *p* = 1.78e-6), and a significant difference between this group and the other two groups in both force-field (g = − 100: t_42_ = 4.51, *p* = 1.52e-4, g = 0: t_42_ = 3.82, *p* = 8.59e-4), and force channel trials (g = − 100: t_42_ = 2.81, *p* = 0.02, g = 0: t_42_ = 2.49, *p* = 0.03, Fig. 9d). This shows that the modulation part of both the predictive and reactive components was higher for the group with skin-stretch in the same direction of the force. Therefore, we conclude that contrary to the manipulation force control, this condition of augmented tactile information augmented the internal representation that is used for grip force control and also caused a reactive increase in grip force.

## Discussion

We studied the effect of augmented tactile information on force-field adaptation. In an adaptation to a velocity-dependent force-field protocol, we exposed participants to one of three conditions of artificial tactile stimulation: skin-stretch in the same direction of the force-field, skin-stretch in the opposite direction, and a control group without artificial skin-stretch. We found that the additional tactile information in either direction did not affect the paths of the participants compared to the control group. In contrast, the augmented tactile information affected both manipulation and grip force control. Adding a skin-stretch in the opposite direction of the force-field increased the adaptation of the internal representation that is used in the control of manipulation forces with respect to adding a skin-stretch in the same direction as the force-field. Interestingly, this stretch did not affect the control of grip force. In contrast, adding a skin-stretch in the same direction of the force-field caused an increase in the predictive and reactive modulation between grip force and load force, and only mild, impeding, effect on manipulation forces. This suggests that tactile information is processed differently for the update of the internal representations that are used for manipulation and for grip force control.

In the current experimental setup, there is an inherent skin deformation in the contact area of the skin with the skin-stretch device, caused by the force that is applied by the kinesthetic haptic device (Fig. 1c). In two of the groups, in addition to this natural stretch of the skin we added artificial skin-stretch, and thus, the different conditions in our study were: (1) additional tactile stimulation in the same direction as the natural stretch, (2) additional tactile stimulation that is opposite to the natural stretch, and (3) without additional tactile stimulation. The current design of our device does not allow for measuring the magnitude of the natural stretch, nor does it allow for measuring the actual extent of the artificial stretch (compared to partial slips of the tactor relative to the skin). Therefore, here we examined the general effect of augmenting the tactile information with a skin-stretch device on force-field adaptation, and determined qualitative differences across directions of stimulation. In future studies, it would be interesting to design a device that can measure the amount of actual skin-stretch, such as the device in [53, 54], and develop a detailed model for the effect of stretch as well as slip signals on force-field adaptation.

### The effect of augmented tactile information on force-field adaptation – control of manipulation force

The augmented tactile information did not affect the paths of the movements. This finding is in line with a recent study that examined the effect of cutaneous information on adaptation to a viscous force-field [19]. In this study, the authors found only a small effect in the beginning of adaptation, and only when the cutaneous information was coupled with the kinesthetic information. However, different adaptation mechanisms may result in similar path error adaptation curves. For example, the adaptation could have been composed from an update of an internal model [11, 12], increasing the impedance of the arm [26, 27], or a gain modulation of reflexes and feedback mechanisms [31–33]. Therefore, to get a more complete understanding of the effect of tactile augmentation on motor adaptation it is important to investigate additional aspects of adaptation, such as our analysis of manipulation and grip force in the current study.

Contrary to our hypotheses, the skin-stretch in the same direction as the force-field caused participants to apply manipulation forces that are less similar to the load forces compared to skin-stretch in the opposite direction. Previous studies showed that adding a skin-stretch in the same direction of the force can augment the perception of stiffness [4, 55, 56], friction [40, 41] and forces [57]. Based on these studies, we expected that adding the skin-stretch will augment the perceived viscosity of the perturbing force-field, and as a result, increase the manipulation forces and lead to faster and more complete learning. However, the artificial skin-stretch as well as partial slips that may occur during augmented tactile stimulation could have also increase the participants’ uncertainty about the perturbing forces, and lead to a co-contraction of their arm muscles. Moreover, following exposure to a skin-stretch in the same direction, the participants applied larger grip forces, consistently with previous studies [4, 44], and larger grip forces are also associated with larger arm impedance [58, 59]. Therefore, a possible explanation to our results is that an increased muscle co-contraction [60] in the group that received skin-stretch in the same direction as the force-field could have reduced their path error and impeded the construction of an internal representation of the force-field for the control of manipulation forces. However, this explanation can be ascertained only in future studies by direct measurement of impedance [28, 59] or assessment of co-contraction from EMG recordings [61].

Why would skin-stretch in the opposite direction of the force-field improve adaptation? Several studies demonstrated that augmented sensory input can enhance motor learning [62–64]. While in most of these studies the visual feedback was manipulated for facilitating motor learning [63, 64], there is nevertheless evidence that also auditory, haptic, and multimodal feedback can affect learning [65–70]. Furthermore, contextual cues can be used to recall a recently learned motor skill [71]. Finally, a recent study reported that cutaneous information can be as effective as kinesthetic in guidance [69]. In our study, the skin-stretch in the opposite direction to the force-field was actually in the direction of the manipulation forces that the participants needed to apply to resist the force-field and to return to a straight path. Hence, this augmented tactile information may have been used as an assistive guidance cue for the task, which enabled participants to learn the perturbation faster. We conclude that adding a skin-stretch in the opposite direction to the force-field might be more suitable for facilitating the construction of a representation of the perturbing force.

It is important to note the large inter-participant variability in our results. This large variability is consistent with previous studies that examined the effect of skin-stretch on perception [4, 42, 56]: participants demonstrated a variety of perceptual responses to stretching their finger pad in different directions. This variability might stem from the difference in participants’ mechanical skin properties [72], different finger sizes [73], small differences in the way they held the device, and many other factors. Nevertheless, even with this large variability, we determined the average effects of augmented tactile information on force-field adaptation. Future studies with larger samples of healthy individuals and patients populations might shed more light on the sources of inter-participant variability in force-field adaptation.

### The effect of augmented tactile information on force-field adaptation – control of grip force

Over the years, studies investigated the role of tactile information in grip force control. These studies showed that the mechanoreceptors in the skin convey information about slippage and movement direction of the object [74], and play an important role in adapting the ratio between grip force and load force to the friction between the object and the skin [4, 34, 36, 75]. In line with a previous study that showed an increase in the grip force-load force ratio when adding a skin-stretch in the context of interaction with elastic objects [4], here we found that skin-stretch in the same direction of the force increased the applied grip force per amount of load. This increase was due to increase in both predictive and reactive components of the modulation of grip force with load force, and not due to a nonspecific increase in the baseline grip force. Surprisingly, and in contrast to our hypotheses, skin-stretch in the opposite direction did not affect the grip force compared to the grip force that participants in the control group applied.

Several studies showed that when exposed to novel load forces, manipulation and grip forces are adjusted in a different manner. Manipulation force control is mainly based on the estimation of the averaged external load forces [76, 77]. In contrast, the predictive grip force control is highly sensitive to load variability [6], and is primarily operated to maintain a consistent GF/LF ratio with an additional safety margin to prevent slippage [22, 23]. Moreover, adaptation of manipulation forces that contribute to adaptation of trajectory relies on kinematic errors, whereas adaptation of grip forces relies on kinetic errors [7]. Our study provides additional evidence that different internal representations are developed throughout the interaction with novel dynamics for the control of manipulation and grip force by showing that the augmented tactile information affects each representation differently.

### Haptics for rehabilitation

We found that augmenting the tactile information with artificial stretch of the skin during force-field adaptation affects the rate and extent of adaptation in the control of manipulation and grip forces. The direct implications of this study are on the basic understanding of the contribution of somatosensory information to force-field adaptation. Nevertheless, it has also potential implications in neurorehabilitation. Augmenting tactile information by means of tactile stimulation devices similar to the one that we used in this study presents a promising avenue for rehabilitation research. Robotic devices have been widely used to facilitate recovery of motor functions [78–81]. Most of these devices apply forces on the patients and stimulate both the kinesthetic and tactile modalities. However, kinesthetic haptic devices are often large, heavy, and expensive, whereas tactile devices are small, lightweight, low cost, and can be wearable [82]. These qualities make tactile devices, combined with virtual reality [83], attractive for ambulatory [84] and in-home rehabilitation [85].

An important issue in physical interaction with robotic devices is stability – it is critical for the safety of the interaction. Regarding to kinesthetic haptic devices, the effectiveness of robotics for rehabilitation may be limited due to the stability constraints [86–88]. Tactile devices do not apply net forces on the users, and therefore, they do not entail instability. Indeed, tactile information was shown to be effective in substituting and augmenting force feedback in teleoperation under stability constraints [89–91]. Our results suggest that a similar approach may be effective in rehabilitation. We expect that additional tactile stimulation opposing the natural stretch will facilitate effective robotic rehabilitation in assistive and resistive robotic interventions. Moreover, using skin-stretch in the same direction as the natural stretch can be used for increasing the applied grip-force and improving the modulation between grip force and the external load that is important for efficient manipulation of objects. This can improve daily activities in pathological cases such as stroke, hand or spinal cord injury that result in reduced hand function [92, 93].

Many neurological disorders, including stroke, entail in addition to motor impairments also somatosensory impairments, including an impairment in tactile sensation, stereognosis and proprioception [94–96]. Somatosensory impairments can significantly affect daily life, and may be the underlying mechanism behind apparent motor impairments. However, most research has focused on the recovery of impaired motor function [78, 97–99], and the somatosensory function received less attention [84]. Previous studies used vibrotactile information to augment healthy and impaired somatosensation either by communicating error or state information via vibration [100–102]. In addition, skin brush information was used to convey directional information as part of efforts to substitute proprioception [84]. If indeed tactile augmentation in the opposite direction to the natural stretch improves force-field adaptation via a high-level guidance information, it is possible that such stimulation will facilitate the recovery of patients with impaired kinesthetic sensing by high-level substitution for the missing information. If this is indeed the case, patients with impaired tactile sensing may also benefit from the stimulation if the information is communicated via an unaffected limb or other area of the body with preserved somatosensation.

Our results suggest that augmenting kinesthetic information with artificial tactile information can affect sensorimotor adaptation, and as such may be useful in rehabilitation. However, studies with each specific solution need to be performed on the target populations before making conclusions about the efficacy of the tactile stimulation that we studied here in neurorehabilitation. For example, it is to be determined in future studies if persons with different neurological disorders integrate kinesthetic and tactile information similarly to healthy individuals. The answer to this question will depend on the disorder, the impairment, and the degree of its severity. Moreover, the device that we used in this paper requires holding the skin-stretch device in a precision grip. This is not appropriate for rehabilitation, where in most of the cases, such fine manipulation ability is heavily impaired. Therefore, an additional research is required to examine the effect of augmented tactile information on force-field adaptation with devices that are robust and do not require precision grip, such as the device in [84] or [103].

## Conclusions

In this study, we examined the effect of augmented tactile information on manipulation and grip force control during adaptation to force-field in healthy individuals. We show that adding a skin-stretch in the same direction of the force-field caused slower adaptation to the force-field in terms of manipulation force control, but increased the modulation between grip force and load force. In contrast, skin-stretch in the opposite direction to the force-field improved the adaptation and did not affect the applied grip forces. These results are important for understanding the effect of tactile information on motor adaptation, which can help in the future in developing efficient haptic devices for assistance and rehabilitation.

## Data Availability

The SolidWorks parts of the skin-stretch device, all the MATLAB code, and the data that was used for the analysis is available at: https://www.dropbox.com/home/Lab%20Stuff/Skin-stretch%20study The authors will be happy to answer any question regarding the presented work by e-mail.

## Abbreviations

EA: Early Adaptation
EW: Early Washout
GF: Grip Force
LA: Late Adaptation
LB: Late Baseline
LF: Load Force
MF: Manipulation Force
